# Interleukin-17–targeted treatment in patients with spondyloarthritis and associated cardiometabolic risk profile

**DOI:** 10.3389/fimmu.2023.1203372

**Published:** 2023-07-18

**Authors:** Rubén Queiro, Elena Aurrecoechea, Sara Alonso Castro, Ignacio Villa Blanco, Anahy Brandy-Garcia, Raquel Linge

**Affiliations:** ^1^Rheumatology and Health Research Institute of the Principality of Asturias (ISPA) Translational Immunology Division, Hospital Universitario Central de Asturias, Oviedo, Spain; ^2^Oviedo University School of Medicine, Oviedo, Spain; ^3^Rheumatology Division, Hospital de Sierrallana, Torrelavega, Spain; ^4^Fundación Instituto de Investigación Marqués de Valdecilla (IDIVAL), Santander, Spain; ^5^Rheumatology Division, Hospital Universitario Central de Asturias, Oviedo, Spain; ^6^Rheumatology Division, Hospital Universitario de Cabueñes, Gijon, Spain; ^7^Novartis Farmaceutica SA, Barcelona, Spain

**Keywords:** biologic therapy, cardiometabolic comorbidities, interleukin-17A, metabolic syndrome, obesity, secukinumab, spondyloarthritis, psoriatic arthritis

## Abstract

Spondyloarthritis is a group of immune-mediated rheumatic disorders that significantly impact patients’ physical function and quality of life. Patients with spondyloarthritis experience a greater prevalence of cardiometabolic disorders, such as obesity, hypertension, dyslipidemia and diabetes mellitus, and these comorbidities are associated with increased spondyloarthritis disease activity and risk of cardiovascular events. This narrative review summarizes the evidence for a physiological link between inflammatory status and cardiometabolic comorbidities in spondyloarthritis, as well as the impact of interleukin (IL)-17 blockade versus other molecular mechanisms in patients with cardiometabolic conditions. The IL-23/IL-17 axis plays a pivotal role in the pathophysiology of spondyloarthritis by promoting inflammation and tissue remodeling at the affected joints and entheses. The importance of the IL-23/IL-17 signaling cascade in underlying sub-clinical inflammation in common cardiometabolic disorders suggests the existence of shared pathways between these processes and spondyloarthritis pathophysiology. Thus, a bidirectional relationship exists between the effects of biologic drugs and patients’ cardiometabolic profile, which must be considered during treatment decision making. Biologic therapy may induce changes in patients’ cardiometabolic status and cardiometabolic conditions may conversely impact the clinical response to biologic therapy. Available evidence regarding the impact of IL-17 blockade with secukinumab on cardiometabolic parameters suggests this drug does not interfere with traditional cardiovascular risk markers and could be associated with a decreased risk of cardiovascular events. Additionally, the efficacy and retention rates of secukinumab do not appear to be negatively affected by obesity, with some studies reporting a positive impact on clinical outcomes, contrary to that described with other approaches, such as tumor necrosis factor blockade. In this article, we also review evidence for this bidirectional association with other treatments for spondyloarthritis. Current evidence suggests that IL-17–targeted therapy with secukinumab is highly effective in spondyloarthritis patients with cardiometabolic comorbidities and may provide additional cardiometabolic benefits.

## 1 Introduction

Spondyloarthritis is a group of immune-mediated rheumatic disorders, including axial spondyloarthritis (axSpA), psoriatic arthritis (PsA), reactive arthritis, enteropathic arthritis and undifferentiated spondyloarthritis (1). These entities are interrelated and share clinical and imaging traits, and a common genetic association with type I major histocompatibility complex HLA-B27 (2). Typical clinical characteristics include inflammation at the sacroiliac joints, spine and entheses, extra-musculoskeletal manifestations (e.g. psoriasis, uveitis and inflammatory bowel disease) and new bone formation at the sacroiliac joints and the spine (3, 4). Axial SpA is characterized by the presence of inflammation along the axial skeleton and can be further divided into ankylosing spondylitis (AS; also known as radiographic axSpA because structural damage in the spine and sacroiliac joints is visible on radiographs) and non-radiographic axSpA (structural damage is not visible on radiographs) (2, 3). PsA is characterized by axial and peripheral musculoskeletal manifestations, accompanied by skin and nail disease (4, 5). PsA and axSpA, as well as other forms of spondyloarthritis, are associated with chronic pain and stiffness, and have a significant impact on patients’ functionality and quality of life (QoL) (2–5).

Although spondyloarthritis disorders are relatively common, their prevalence varies widely across different demographic characteristics, such as age, sex and geographical region, and according to the methodology used in the studies (1). The global prevalence of spondyloarthritis disorders ranges from 0.2% to 1.6%, with AS varying between 0.02% and 0.4%, and PsA between 0.01% and 0.2% (1). In the Spanish EPISER2016 study, the prevalence of AS and PsA was 0.3% and 0.6%, respectively (6, 7).

Treatment goals for spondyloarthritis are disease remission (control of disease activity and prevention of radiographic progression), maintenance of physical function and improvement in QoL (8, 9). First-line treatment for both axSpA and PsA patients is non-steroidal anti-inflammatory drugs (NSAIDs), accompanied by physical therapy and exercise (5, 9, 10). Conventional disease-modifying antirheumatic drugs (DMARDs), such as methotrexate, sulfasalazine or leflunomide, are generally ineffective in the axSpA setting (10), though they are commonly used for peripheral manifestations based on observational data (11).

Biologic therapies targeting the two main inflammatory pathways involved in the development of axSpA and PsA, the tumor necrosis factor alpha (TNF-α) and the interleukin (IL)-23/IL-17 axes, are the most common choice for spondyloarthritis patients whose disease remains active despite treatment with conventional DMARDs (5, 9, 10, 12). The TNF-α inhibitors (TNFis) infliximab, etanercept, adalimumab, golimumab and certolizumab, and the IL-17 inhibitors (IL-17is) secukinumab and ixekizumab are routinely used for the treatment of axSpA and PsA. A new molecule, bimekizumab, which simultaneously targets IL-17A and IL-17F, has also shown positive results in the treatment of axSpA and PsA, and is currently in development (13). The IL-12/23 p40 inhibitor ustekinumab and the IL-23 p19 inhibitors guselkumab and risankizumab are effective therapies for PsA, and other IL-23 p19 inhibitor, tildrakizumab, is being investigated for its therapeutic potential in PsA. However, both the blockade of IL-12/23 and IL-23 have failed to show efficacy in patients with axSpA (14, 15). Other targeted agents include the phosphodiesterase-4 inhibitor (PDE4i) apremilast, and Janus kinase/signal transducer and activator of transcription inhibitors (JAK/STATis; e.g. filgotinib, tofacitinib, upadacitinib and deucravacitinib) (10). Some of these agents are in clinical trials for spondyloarthritis, and some have been approved and already entered the guidelines but are yet to become established in clinical practice (9, 10, 12).

Spondyloarthritis disorders, mainly axSpA and PsA, are frequently associated with a number of cardiometabolic comorbidities, including obesity, hypertension, dyslipidemia and diabetes mellitus, collectively known as metabolic syndrome (MetS), and consequently, with an increased risk of cardiovascular morbidity and mortality (16–18). Therapeutic decisions in spondyloarthritis patients have traditionally relied on evidence from randomized controlled trials confirmed by real-world studies and on the benefit–risk profile of each intervention; however, it is important to consider cardiometabolic comorbidities in treatment decisions, given the high prevalence of these conditions (19). While clinical guidelines for axSpA and PsA recommend considering comorbidities along with active disease when choosing the optimal treatment (20–22), guidelines from the European Alliance of Associations for Rheumatology (EULAR) for cardiovascular disease (CVD) risk management recommend early and aggressive screening for CVD risk factors and MetS components for all patients with inflammatory joint disorders (23).

In this narrative review, we summarize the literature on axSpA and PsA, examining the physiological link between the inflammatory state and cardiometabolic comorbidities, as well as the impact and efficacy of IL-17–targeted therapy in spondyloarthritis patients with cardiometabolic comorbidities.

## 2 Search strategy and selection criteria

We conducted a PubMed search in July 2022 using the search term “spondyloarthritis” alone or in combination with one or more of the following terms: “inflammation”, “cardiovascular risk”, “obesity”, “metabolic syndrome”, “TNF”, “IL-17” and “IL-23”. Potentially relevant articles were chosen based on the title and abstract. Additional references were accessed as needed. Articles within the last 5 years were prioritized, although older articles were considered if relevant to the topic.

## 3 Cardiometabolic profile of spondyloarthritis patients

### 3.1 Prevalence of cardiometabolic comorbidities

There is a high prevalence of cardiometabolic abnormalities among patients with axSpA and PsA (17, 24–28); these abnormalities are characteristic of these spondyloarthritis conditions and distinct from those found in other autoimmune disorders, such as rheumatoid arthritis (RA) (27). A questionnaire-based assessment of cardiovascular risk factors in 692 patients with PsA showed that these patients had a significantly higher prevalence of obesity (28.6% *vs* 16.3%; p<0.001), hypertension (40.3% *vs* 24.1%; p<0.001) and diabetes (10.5% *vs* 6.2%; p<0.001) compared with matched controls (29). In another assessment of 2896 patients with PsA, AS or RA, obesity was more prevalent among those with PsA (23.0%) than in those with AS (17.0%) or RA (15.2%) (30). Similar results have been reported in two retrospective cross-sectional studies by Queiro and colleagues (31, 32). One study showed that PsA patients had a significantly higher prevalence of obesity (35.0% *vs* 22.0%; p<0.0001), hypertension (36.0% *vs* 23.0%; p<0.0001) and diabetes (13.8% *vs* 5.0%; p<0.0001) compared with patients without inflammatory conditions (32). The second study showed a significantly higher prevalence of obesity both in patients with PsA (27.6% *vs* 22.0%; p<0.05) and psoriasis (36.5% *vs* 22.0%; p<0.01) compared with matched controls (31). A comparison between patients with PsA and psoriasis showed that obesity (odds ratio [OR] 1.77; 95% confidence interval [CI] 1.23–2.56; p=0.002) (33) and hyperlipidemia (OR 2.5; 95% CI 1.7–3.3; p<0.01) (34) were more prevalent in patients with PsA.

An assessment of comorbidities among 202 Chinese patients with spondyloarthritis revealed that 3.0% had diabetes, 20.3% had hypertension and 30.8% had hyperlipidemia (35). Further, analysis of a large cohort of North American patients found that PsA patients were significantly more likely to have MetS than patients with RA (OR 1.44; p=0.02) (36). For the individual MetS components, PsA patients were significantly more likely to have hypertriglyceridemia (OR 1.51; p=0.003) and diabetes (OR 1.56; p=0.02) than RA patients, and non-significant increases in the odds of obesity, hypertension and low levels of low-density lipoprotein cholesterol (LDL-C) were also observed (36). In a Mediterranean population, the prevalence of MetS was significantly higher among patients with PsA than in matched controls (54.8% *vs* 36.6%; p=0.02) (37).

A systematic review and meta-analysis assessing the prevalence of MetS in PsA (24 studies), psoriasis (89 studies) and RA (53 studies) populations showed that PsA patients were more likely to have MetS than those with psoriasis (OR 1.61; 95% CI 1.49–1.73) or RA (OR 1.66; 95% CI 1.54–1.79) (38). Another meta-analysis revealed that the three most prevalent comorbidities in axSpA patients were hypertension, hyperlipidemia and obesity (pooled prevalence of 23%, 17% and 14%, respectively) (39).

The heterogeneity in comorbidities experienced by patients with axSpA and PsA may be due to various reasons, like geographic or lifestyle factors (40–42). In a study comparing the epidemiology of comorbidities between Italian and Belgian patients with PsA, there were differences in comorbidity rates between countries, in particular in the prevalence of hypertension (42). From the results of this study, it can be inferred that countries whose diets are based on the so-called “Mediterranean diet” (Italy, Greece, Spain) may have significantly different rates of obesity and other cardiometabolic risk factors than in other countries (43). Furthermore, a cross-sectional study of the Spanish AtheSpAin cohort, suggested that there were differences between the sexes when it came to comorbidities in patients with axSpA (44). Specifically, there were disease-related features that may influence the formation of atherosclerosis in female patients with axSpA compared with males. Finally, there are different comorbidity profiles seen among patients with SpA (PsA and non-PsA SpA) (45).

### 3.2 Impact of cardiometabolic comorbidities on disease activity

The presence of cardiometabolic abnormalities is linked to PsA and axSpA disease activity and the inability to achieve a minimal disease activity (MDA) status (27). In particular, obesity has been associated with inflammation, disease activity and cardiovascular risk factors (46–50). A systematic literature review assessing radiological outcomes of spondyloarthritis patients showed that obesity was associated with entheseal inflammation and both axial and peripheral new bone formation (51). In AS patients, an increase in Ankylosing Spondylitis Disease Activity Score (ASDAS) of 0.06 (95% CI 0.04–0.08) units has been reported with every 1 kg/m^2^ increase in body mass index (BMI) (47). Furthermore, significantly higher C-reactive protein (CRP) levels, disease activity (assessed by ASDAS-CRP), radiographic damage and impact on physical mobility, liver function and blood pressure (all p<0.05) have been reported in obese patients with AS (49).

A study in Norwegian axSpA patients from the European Map of Axial Spondyloarthritis survey revealed a significantly higher disease activity (measured by the Bath Ankylosing Spondylitis Disease Activity Index [BASDAI]) in obese patients compared with normal or underweight patients (mean ± standard deviation [SD] 5.87 ± 1.78 *vs* 4.99 ± 2.08; p<0.001) (48). Moreover, overweight and obese patients reported a significantly greater degree of spinal stiffness compared with normal or underweight patients (p<0.001 and p=0.006, respectively) (48). Additionally, another study in axSpA patients found that obesity was an independent predictor of worse clinical outcomes and reduced QoL (p<0.05) (52). These findings are supported by a systematic review and meta-analysis that demonstrated that overweight and obese patients with axSpA tend to present with higher disease activity scores than normal-weight patients (53). These differences appeared to be clinically meaningful for the comparison between obese patients and patients with a normal BMI, and especially when disease activity was assessed using BASDAI rather than ASDAS (53).

Although the association between obesity and disease activity in spondyloarthritis patients is well known, studies assessing the effects of weight loss are scarce. To date, two studies have demonstrated the correlation between weight loss and achievement of MDA in overweight and obese patients with PsA (54, 55). In one study, obese PsA patients on TNFi therapy were significantly more likely to achieve MDA while on a hypocaloric diet versus a free-managed diet (54). In the second study, obese PsA patients who underwent weight loss management with a very low energy liquid diet for 12–16 weeks followed by a gradual reintroduction of energy restricted diet, displayed significant improvements in disease activity after 6 months of follow-up (55), which were sustained for up to 2 years with concurrent improvements in cardiovascular risk factors (56). The proportion of patients with MDA increased from 28.2% at baseline to 38.5% at 12 months (p=0.008) and 45.7% at 24 months (p=0.016) (56).

### 3.3 Inflammatory pathways shared between cardiometabolic disorders and spondyloarthritis: the role of interleukin-17

The high prevalence of cardiometabolic abnormalities among PsA and axSpA patients and their link with disease activity suggests the existence of shared signaling pathways and interdependent inflammatory mechanisms. Indeed, the immune system and metabolism are linked through a network of soluble mediators, such as adipokines and cytokines (24, 25, 57).

**Figure 1:**
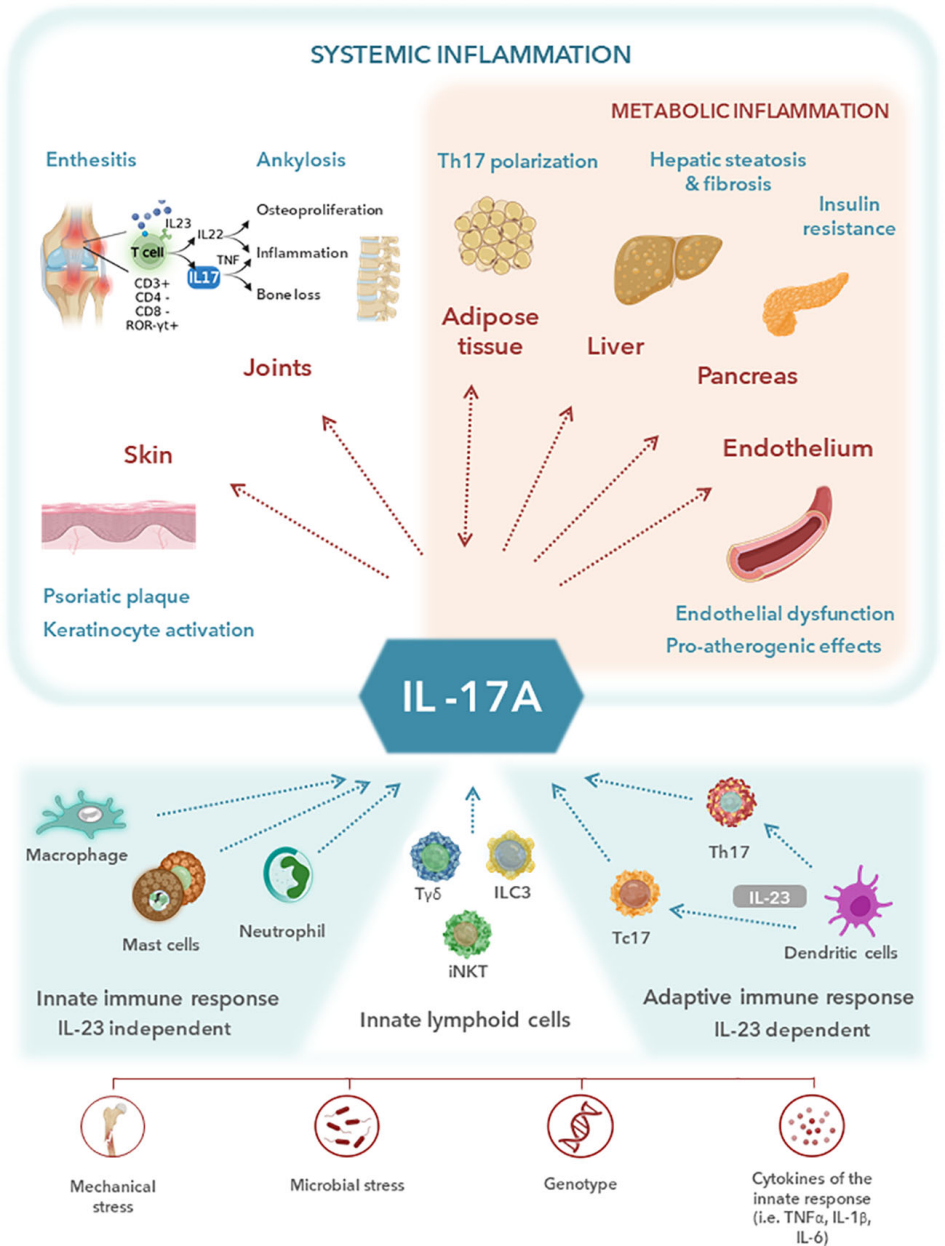
Central role of interleukin (IL)-17A in the musculoskeletal and metabolic manifestations of systemic inflammation. IL-17A plays a central role in the inflammatory pathways shared between cardiometabolic disorders and spondyloarthritis (SpA). IL-17A is primarily produced by T helper 17 (Th17) cells, whose activation, proliferation and survival is driven by IL-23. Other cellular sources of IL-17A include adaptive immune cells and innate immune cells such as type 3 innate lymphoid cells [ILC3], γδ T cells, and invariant natural killer T cells [iNKT] among others. Despite the important role of IL-17A in promoting inflammation and host defence against specific pathogens, increased IL-17A expression is known to be involved in the pathophysiology of several chronic inflammatory diseases, including psoriatic arthritis (PsA) and axial SpA (axSpA). Metabolic disorders, which are common comorbidities in SpA patients, are known to be mechanistically correlated with a chronic inflammatory status. IL-17A has been identified as a crucial contributor to several pathological processes leading to metabolic syndrome. Upregulation of IL-17A expression in PsA and axSpA patients may be a key factor in the metabolic-related inflammatory profile, explaining the high prevalence of cardiometabolic comorbidities among SpA patients. TNF, tumor necrosis factor.

IL-17 comprises a family of six pro-inflammatory cytokines (A to F), of which IL-17A is the best described and most potent member (58–60). IL-17A is primarily produced by T helper 17 (Th17) cells, a specific type of CD4+ T cell, whose activation, proliferation and survival is driven by IL-23 (59, 61–63). Other cellular sources of IL-17A include innate immune cells (e.g. type 3 innate lymphoid cells [ILC3], αβ and γδ T cells, mucosal-associated invariant T cells [MAIT] and invariant natural killer T cells [iNKT]) and adaptive immune cells (e.g. IL-17+/CD8+ T cells and tissue-resident memory [T_RM_] T cells) (**Figure 1**) (58, 60–66). IL-17A acts on several cellular targets, including keratinocytes, neutrophils, endothelial cells, osteoclasts, chondrocytes and osteoblasts, and stimulates the production of antimicrobial peptides, chemokines and pro-inflammatory cytokines (58, 60–62, 65). Under physiological conditions, IL-17A promotes tissue repair on mucosal surfaces and supports anti-infective immune responses (**Figure 1**) (58, 62, 64).

Despite the important role of IL-17A in promoting inflammation and host defense against specific pathogens, increased IL-17A expression is known to be involved in the pathophysiology of several chronic inflammatory diseases, including PsA and axSpA (66). The pivotal role of IL-17 in the development of PsA and axSpA has been demonstrated in several studies. Mast cell infiltration and IL-17A expression in synovial inflammation (61, 62, 65) and elevated serum levels of IL-17A and IL-23 (66) have been described in patients with spondyloarthritis. PsA patients have increased Th17 cells in the blood and synovial fluid (61), with the number of synovial Th17 cells being correlated with CRP levels, erythrocyte sedimentation rate and Disease Activity Score 28 (DAS28) (62). Studies in patients with PsA have also found high levels of IL-23, IL-17A and IL-17 receptors in synovial membranes and overexpression of IL-17 (and IL-22) in entheses resident Th17 cells (61, 66). Patients with AS have a high number of IL-17-secreting cells in the facet joints (higher than in osteoarthritis patients) and increased serum IL-17 levels compared with healthy individuals, with serum IL-17 levels and disease activity (as measured by the BASDAI index) having a significant positive correlation (58, 62, 66). In addition, several single-nucleotide polymorphisms have been identified in genes directly involved in IL-17 signaling in patients with PsA or AS (64).

Activation of the IL-23/IL-17 axis in spondyloarthritis patients triggers joint inflammation and tissue remodeling through several mechanisms. Neutrophil recruitment to joint spaces is promoted by the production of granulocyte-colony stimulating factor, granulocyte-macrophage stimulating factor and chemokines (61). Stimulation of the IL-23/IL-17 axis also promotes angiogenesis, thereby facilitating the influx of inflammatory cells into the inflamed tissues (66). Other mechanisms include upregulation of the transcription of genes that promote the secretion of bone matrix-degrading enzymes, including matrix metalloproteinase-9, tartrate-resistant acid phosphatase and cathepsin K (61, 66), stimulation of bone-resorbing osteoclast differentiation (66, 67), and promotion of osteoblast differentiation and entheseal bone formation (67).

**Figure 2:**
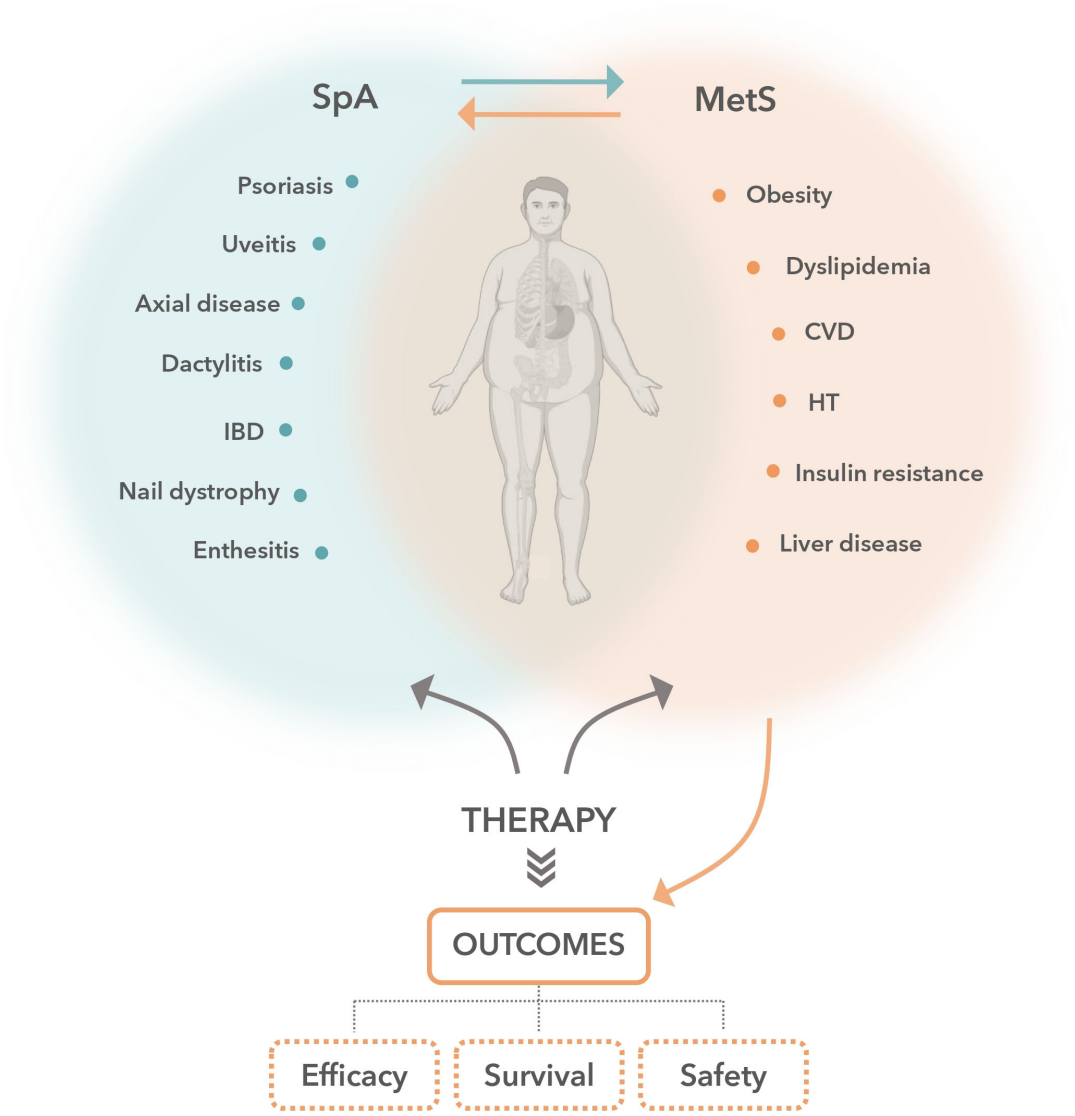
Crosstalk between cardiometabolic conditions and spondyloarthritis treatment. There is a bidirectional relationship between the effects of biologic disease-modifying antirheumatic drugs and the patient’s cardiometabolic profile that should be considered during therapeutic decision-making in patients with spondyloarthritis (SpA). SpA disorders are associated with an increased prevalence of cardiometabolic comorbidities, such as obesity, hypertension, dyslipidemia and diabetes mellitus, collectively known as metabolic syndrome (MetS) and thus with an increased risk of cardiovascular morbidity and mortality. On the one hand, patient cardiometabolic conditions have a notable impact on SpA treatment outcomes, modifying the probability of achieving inactive disease status, the treatment persistence or the pattern of adverse events occurrence. On the other hand, SpA therapies can differentially alter patient cardiometabolic parameters and cardiovascular risk. Therefore, considering this interplay and how each treatment option influences the above in a distinct manner is critical to selecting the best treatment strategy. CVD, cardiovascular disease; HT, hypertension; IBD, inflammatory bowel disease.

Metabolic disorders, which are common comorbidities in spondyloarthritis patients, are known to be mechanistically correlated with a chronic inflammatory status (**Figure 2**). A growing body of evidence suggests that upregulation of the IL-23/IL-17 axis in PsA and axSpA patients may be a key factor in the metabolic-related inflammatory profile, explaining the high prevalence of cardiometabolic comorbidities among spondyloarthritis patients. Obese individuals have higher serum IL-23 and IL-17 levels than non-obese individuals (68–70) and increased IL-17 levels have been found in patients with type 1 and 2 diabetes, non-alcoholic fatty liver disease or steatohepatitis (69). Moreover, the IL-17 receptor levels in liver and muscle cells have been correlated with insulin resistance in obese patients (71). In addition, infiltrating CD4+ T cells, including Th17 cells, have been shown to promote a pro-inflammatory environment and insulin resistance in adipose tissue in obese individuals (72). IL-17A also appears to be involved in the development of fatty liver disease, with evidence suggesting that the IL-17 axis plays a crucial role in the pathogenesis of steatohepatitis and progression to fibrosis (73, 74).

Evidence also suggests that IL-17 affects the cardiovascular system. IL-17A promotes endothelial dysfunction and oxidative stress (75), induces apoptosis of the endothelial cells and cardiomyocytes (63), downregulates the expression of pro-adipogenic transcription factors and upregulates the expression of anti-adipogenic factors (68) and accelerates atherosclerosis (63).

In summary, IL-17 plays a homeostatic role in promoting inflammation and host defense against specific pathogens (e.g. maintenance of the barrier functions of the intestinal epithelium, defense against bacteria and fungi, etc.), but at the same time, depending on the pathology and its location, it also exerts clearly pro-inflammatory actions, for example at the cutaneous or enthesitis level, as well as playing a role in the cardiovascular system.

## 4 Therapies targeting interleukin-17: beneficial effects beyond disease control

Data described above suggest that spondyloarthritis treatments that target IL-17, and the consequent decrease of inflammation, may also help in controlling cardiometabolic comorbidities. In this section, we will explore the impact of IL-17is on cardiometabolic variables, as well as their efficacy and treatment persistence in patients with cardiometabolic abnormalities, and compare them with other biologic DMARDs, including TNFis (the most widely used treatment for spondyloarthritis), IL-12/23is and PDE4is. Inhibitors of other molecular targets such as JAK/STATis have been recently approved for the treatment of spondyloarthritis (76); however, there is still no evidence on how cardiometabolic comorbidities impact their efficacy and persistence. Safety profile of JAK/STATis in patients with increased cardiovascular and venous thromboembolism risk factors is currently being reviewed, due to evidence of possible increases in cardiovascular and thromboembolic events and therefore they should be used with caution in this patient population (21, 77, 78).

### 4.1 The impact of interleukin-17–targeted treatments on cardiometabolic parameters

Studies indicate that IL-17–targeted therapy may reduce markers of inflammation and cardiovascular risk. In a pooled analysis of 19 clinical trials of secukinumab in patients with psoriasis, PsA and axSpA, a rapid reduction in inflammatory markers (high-sensitivity CRP and neutrophil-lymphocyte ratio) occurred after 12–16 weeks of treatment, whereas all traditional cardiovascular risk factors, such as BMI, fasting glucose, blood pressure, LDL-C, total cholesterol (TC), high-density lipoprotein cholesterol (HDL-C) and triglycerides (TG), remained stable after 1 year (79). A study in PsA patients showed a significant decrease in some adipocytokines (resistin and chemerin) with secukinumab in male but not female patients, and no differences in adiponectin or CRP levels within the first 6 months of treatment (80).

An observational study examined the efficacy of different biologic agents (e.g. TNFis [adalimumab, etanercept], IL-12/23i [ustekinumab] and IL-17is [secukinumab, ixekizumab]) compared with non-biologic therapy for reducing inflammation-driven phenotypes of coronary plaque in patients with severe psoriasis (81). After 1 year of treatment, there was a 6% reduction in non-calcified coronary plaque burden (p=0.005) and a significant improvement in plaque morphology (57% reduction in necrotic burden and 55% reduction in fibro-fatty burden) with biologic therapy (n=89) versus non-biologic therapy (n=32). Although all biologic agents were associated with significant reductions in non-calcified plaques, the greatest percentage reduction was seen in patients treated with IL-17is, indicating a potential role of IL-17is in reducing cardiovascular risk (81). It is important to note, however, that this comparison was not adjusted for confounding, and that the study had several limitations, as it was an observational study where biologic agents were given in an open-label, non-randomized manner to a small sample of patients with short duration of follow-up. As such, these results must be confirmed in a randomized clinical trial. Other authors have reported reductions in the intima-media thickness of patients with severe psoriasis treated with an IL-17A inhibitor (82). In line with these findings, the Evaluation of Cardiovascular Risk Markers in Psoriasis Patients Treated with Secukinumab (CARIMA study) demonstrated that flow-mediated dilation was significantly higher than baseline in patients receiving the 300 mg dose of secukinumab for 52 weeks (+2.1%; 95% CI 0.8–3.3; p=0.0022). This result suggests that secukinumab might have a beneficial effect on cardiovascular risk by improving the endothelial function of patients with psoriasis (83).

The impact of IL-17 targeted treatments on cardiovascular risk markers has been mainly studied in patients with psoriasis. However, since psoriasis and PsA (the so-called psoriatic disease) share pathophysiological mechanisms that promote increased cardiovascular risk, it is potentially feasible that these effects may also occur in PsA (or even axSpA). Further research is warranted to confirm these effects in SpA.

### 4.2 The impact of other biologic treatments on cardiometabolic parameters

Several studies have evaluated the impact of various biologic DMARDs on cardiometabolic parameters and cardiovascular risk in patients with PsA or psoriasis. In a cohort study in patients with psoriasis or PsA, there was no significant difference between ustekinumab and TNFi therapy in the risk of atrial fibrillation (adjusted hazard ratio [HR] 1.08; 95% CI 0.76–1.54) or major adverse cardiovascular events (MACE; adjusted HR 1.10; 95% CI 0.80–1.52) (84). In contrast, another cohort study in PsA patients who were new users of biologic DMARDs (TNFis, IL-23is, IL-17is or PDE4is) showed a greater risk of MACE with IL-23i therapy (HR 2.0; 95% CI 1.3–3.0) or IL-17i therapy (HR 1.9; 95% CI 1.2–3.0) compared with TNFis, but this risk did not significantly differ between PDE4i and TNFi therapy (85). However, this study may have been subject to certain bias, as the results were not adjusted for confounding factors such as disease severity, obesity, smoking status, family risk factors or NSAID use (85). Therefore, this result might be explained on the basis of confounding by indication, accounting for the fact that IL-12/23 and IL-17 inhibitors were administered to patients who were already at a higher risk of MACEs, as suggested by the greatest number of morbidly obese or diabetic patients in these groups.

There is conflicting evidence regarding the impact of TNFis on the lipid profile and the risk of cardiovascular events, with some studies showing a cardioprotective effect of TNFis and others reporting no significant effect (86–91). In a cohort study of 238 axSpA patients, of whom 132 were receiving TNFis, there was no significant difference in the atherogenic lipid profile (TC, TG, LDL-C and HDL-C) of TNFi users when compared with TNFi non-users after 2 years of treatment (with the exception of a small but significant increase in TC from 177.86 ± 28.73 to 183.08 ± 29.83 mg/dL; p=0.019) (92). Another study found that TNFi therapy was associated with significant improvements in brachial artery flow-mediated vasodilation at 6 months and pulse-wave velocity at 12 months in patients with AS or RA, whereas no changes were detected in the common carotid intima-media thickness (93). However, no changes in the lipid profile of the same patient cohort were reported after 1 year of TNFi treatment (94). A retrospective study in axSpA patients reported reduced cardiovascular risk with TNFi therapy, but this association was non-significant after adjusting for the erythrocyte sedimentation rate and CRP levels (adjusted HR 0.37; 95% CI 0.12–1.12; p=0.077), suggesting that the observed reduction was due to inflammation control and not due to any TNFi-specific effect (95).

The risk of cardiovascular events was reduced in a cohort study of patients with AS, PsA or RA among those treated with TNFis (HR 0.85; 95% CI 0.76–0.95), as well as in those who received other biologic DMARDs (abatacept, anakinra, rituximab or tocilizumab; HR 0.81; 95% CI 0.70–0.95), but there was no risk reduction in patients who had ceased biologic therapy (HR 0.96; 95% CI 0.83–1.11) (96). Another study that evaluated the risk of myocardial infarction, stroke and revascularization in PsA patients treated with TNFis or other biologics showed low incidence rates of cardiovascular events with all biologics, but the incidence rate of myocardial infarction was particularly low in TNFi-treated patients (1.4 per 1000 persons-year; 95% CI 1.0–1.8) (97).

In a systematic review and meta-analysis of nine observational studies in AS patients, there was no significant association between TNFi therapy and the incidence of myocardial infarction (relative risk [RR] 0.88; 95% CI 0.57–1.35); however, the level of the evidence for this analysis was low due to the observational nature of the included studies (98). Another systematic review and meta-analysis showed a decreased risk of cardiovascular events among patients with psoriasis or PsA who were treated with antirheumatic drugs, including TNFis (RR 0.75; 95% CI 0.63–0.91; p=0.003) (99). An updated meta-analysis on the effect of TNFis on adverse cardiovascular events in psoriasis patients (with or without PsA) revealed a significantly lower cardiovascular risk with TNFis versus topical/phototherapy (RR 0.58; 95% CI 0.43–0.77; p<0.001) or methotrexate (RR 0.67; 95% CI 0.52–0.88; p=0.003) (100).

Evidence of the effect of TNFis on cardiometabolic parameters suggests that TNFi treatment has a negative impact on body composition, weight and BMI, and is generally associated with weight gain and an increase in fat mass (namely android fat mass) (101–103). In spondyloarthritis patients, 2 years of TNFi therapy was associated with significant increases from baseline in mean ± SD BMI and fat mass of 0.7 ± 1.8 kg/cm^2^ (p<0.05) and 0.7 ± 1.0 kg (p<0.001), respectively (101). Moreover, these patients also had significant gains from baseline in mean ± SD waist circumference (of 4.1 ± 5.9 cm; p<0.001), visceral adipose tissue (of 29.1 ± 33.4 cm^2^; p<0.001) and subcutaneous adipose tissue (of 1.9 ± 53.2 cm^2^; p<0.001) (101). Similarly, a study of patients with RA or AS observed increases from baseline in body weight (1.9%; p=0.003), BMI (2.5%; p=0.004), total fat mass (11.1%; p=0.007) and fat in the android region (18.3%; p=0.02) after 2 years of TNFi treatment (102). In psoriasis patients treated with TNFis (etanercept and infliximab) or methotrexate for 6 months, significant gains in weight and BMI from baseline were observed in patients treated with TNFis, whereas non-significant changes were observed in patients treated with methotrexate (104). In this study, patients treated with TNFis were 4.3 times more likely to gain ≥5 kg of body weight than those receiving methotrexate (104). In a study of spondyloarthritis patients, simultaneous increases in serum insulin-like growth factor-I (IGF-I; 15% increase from baseline, p=0.04), body weight, lean mass and bone mineral density were observed after 3 months of TNFi treatment (103).

### 4.3 Efficacy and persistence of interleukin-17 inhibitors in patients with cardiometabolic comorbidities

In a prospective study of secukinumab-treated PsA patients who were overweight/obese (BMI ≥25 kg/m^2^) or normal weight (BMI <25 kg/m^2^), obese patients had higher circulating secukinumab concentrations compared with normal-weight patients after 6 months of treatment (105). Moreover, this study found a significant, although relatively small (rho 0.1), inverse correlation between disease activity (assessed by the Disease Activity Index for Psoriatic Arthritis [DAPSA]) and BMI, suggesting that overweight and obese patients may have had a better response to secukinumab treatment than normal-weight patients (105).

Real-world data indicate that secukinumab retention rates are not negatively influenced by cardiometabolic comorbidities. In the observational SEcukinumab in Cantabria and ASTURias (SECASTUR) study in patients with axSpA or PsA, the 1-year retention rate with secukinumab was 66% in a population largely refractory to biologic therapy (106). Of note, patients with obesity (HR 0.53; 95% CI 0.30–0.93; p=0.027), hypertension (HR 0.55; 95% CI 0.35–0.93; p=0.008) or diabetes (HR 0.42; 95% CI 0.18–0.99; p=0.047) had a significantly lower risk of secukinumab treatment discontinuation (106). In another real-world study of Spanish patients with PsA or axSpA, the secukinumab retention rate was 71% after 1 year of treatment; the best retention rates were seen in women with axSpA and men with PsA (107). In this study, obesity did not affect the secukinumab retention rate (HR 2.54; 95% CI 0.99–6.50; p=0.051 *vs* non-obese patients); in fact, obese women had a significantly higher probability of treatment persistence (HR 0.046, 95% CI 0.005–0.44; p=0.007 *vs* non-obese women).

The ongoing non-interventional AQUILA study is investigating the real-world effectiveness and safety of secukinumab treatment in 3000 patients with active PsA (108) or AS (109) according to their BMI. In patients with PsA, preliminary reports described improvements in disease activity (e.g. Psoriatic Arthritis Impact of Disease-12 [PsAID-12] score and Patient’s Global Assessment [PGA]) up to 1 year in all BMI subgroups (108). Similarly, in patients with AS, preliminary reports described improvements in disease activity (e.g. BASDAI) and in global functioning and health (Assessment of Spondyloarthritis international Society Health Index [ASAS–HI]) up to 1 year in all patients, irrespective of their BMI (109). Moreover, secukinumab treatment had a favorable safety profile, with no new safety concerns identified in these patients (108, 109).

There currently appears to be no evidence of how cardiometabolic conditions affect ixekizumab and bimekizumab outcomes in spondyloarthritis.

### 4.4 Efficacy and persistence of other biologic treatments in patients with cardiometabolic comorbidities

While the presence of obesity appeared to improve or had no effect on secukinumab efficacy and treatment persistence, studies suggest that obese patients may have a poor response to ustekinumab or TNFis and an increased likelihood of treatment discontinuation. Real-world data from a Spanish cohort of patients with psoriasis or PsA showed a ustekinumab retention rate of 62% after a mean follow-up of 28.14 ± 24.02 months, but the retention rate was significantly lower among obese patients (p=0.0001), with 63.6% of obese patients discontinuing ustekinumab and 13.9% continuing treatment (110). However, ustekinumab dose adjustment according to weight was not always followed, which may have biased the results in this study (110).

The efficacy and retention rate of TNFis in patients with cardiometabolic abnormalities have been explored in several studies, in which obese patients had a poorer response and were more likely to discontinue treatment than normal-weight patients (111–113). In a real-world cohort study of Swiss axSpA patients who received TNFi as first-line therapy, obese patients had significantly lower odds of achieving 40% improvement in the Assessment in SpondyloArthritis international Society (ASAS40) response compared with normal-weight patients (OR 0.27; 95% CI 0.09–0.70) (114). Another real-world study in Chinese AS patients receiving different biologics showed a significant negative correlation between disease activity and BMI among TNFi users after 3, 6, 9 and 12 months of treatment (115). In an Italian cohort of PsA patients, the presence of MetS was associated with a lower probability of achieving MDA after 24 months of TNFi treatment (OR 0.56; 95% CI 0.43–0.72; p<0.001) (116). Results from the DANBIO (Denmark) and ICEBIO (Iceland) registries showed that obese PsA patients treated with TNFis had higher disease activity (i.e. decreased odds of achieving a EULAR good or moderate response [OR 0.47; 95% CI 0.29–0.72]), reduced clinical response and poorer treatment adherence (HR 1.6; 95% CI 1.3–2.0) after 18 months of treatment compared with non-obese patients (117). In contrast, a study by Ianonne and colleagues has reported no significant differences in disease activity (according to DAS28 and the Simple Disease Activity Index [SDAI]) between obese and non-obese PsA patients treated with TNFis (118). This discrepancy may be caused by the retrospective nature of the study and the inclusion/exclusion criteria used to define the study population (118).

The poor clinical response to TNFis among obese patients has also been confirmed in meta-analyses. In a systematic review and meta-analysis, the odds of achieving a good response or clinical remission (50% improvement in BASDAI) with TNFis were lower in obese axSpA patients compared with non-obese patients (OR 0.41; 95% CI 0.21–0.83) (119). In another systematic review of studies in PsA patients treated with TNFis, ustekinumab, abatacept or apremilast, obese patients had a higher likelihood of achieving 20% improvement in American College of Rheumatology criteria (ACR20; OR 1.42; 95% CI 1.00–2.08) but significantly higher odds of treatment withdrawal (OR 1.60; 95% CI 1.34–1.92) than non-obese patients (120).

There is abundant literature on obesity and its deleterious effects on TNFi efficacy in spondyloarthritis patients, but data on the effects of weight loss on disease activity are scarce. In a study in overweight/obese PsA patients treated with TNFi and receiving a dietary intervention, a ≥5% weight loss from baseline was associated with significantly higher rates of MDA achievement at 6 months follow-up compared with <5% weight loss (OR 3.75; 95% CI 1.36–10.36 for patients with 5–10% weight loss and OR 6.67; 95% CI 2.41–18.41 for patients with >10% weight loss) (54). Similarly, another study in obese psoriasis patients treated with TNFis reported that dietary interventions resulted in a significant weight loss (–12.9 ± 1.2 kg *vs* –1.5 ± 0.5 kg) and a significant improvement in disease activity (Psoriasis Area and Severity Index 75 [PASI 75] achieved in 85.9% *vs* 59.3%; p<0.001) at week 24 compared with no dietary interventions (121).

## 5 Conclusions

A bidirectional relationship exists between the effects of biologic DMARDs and patient´s cardiometabolic profile that must be considered during treatment decision making in patients with spondyloarthritis. Biologic therapy may cause changes in patients’ cardiometabolic status, which may conversely impact the clinical response to treatment. Although evidence on the effect of IL-17–targeted drugs on cardiometabolic profile is limited, the available data suggest that secukinumab does not interfere with traditional cardiovascular risk markers and could be associated with a decreased risk of cardiovascular events. Secukinumab is as effective in obese as in non-obese patients, and drug retention rates are high in obese spondyloarthritis patients. While there is limited evidence regarding IL-23–targeted drugs, there are abundant data on the impact of TNFis on the cardiometabolic profile. TNFis are mostly associated with increases in body weight and fat mass, and poorer clinical responses and increased discontinuation rates in obese patients. Although further studies are necessary as no comparative randomized clinical trials are available to date, these data suggest that patients with axSpA or PsA and cardiometabolic disorders may benefit from an IL-17A–targeted therapy rather than other interventions, with secukinumab being the only IL-17i that has currently demonstrated such benefits.

## Author contributions

All named authors meet the International Committee of Medical Journal Editors (ICMJE) criteria for authorship for this article, take responsibility for the integrity of the work as a whole, and have given their approval for this version to be published. RQ, EA, SAC, IVB, AB-G and RL were responsible for the conceptualization and design of the review, critically reviewing and revising all drafts of the review, and approving the final version of the manuscript. All authors contributed to the article and approved the submitted version.

## Glossary

**Table:**

ACR	American College of Rheumatology
AS	ankylosing spondylitis
ASAS	Assessment in SpondyloArthritis international Society
ASAS-HI	Assessment of SpondyloArthritis international Society Health Index
ASDAS	Ankylosing Spondylitis Disease Activity Score
axSpA	axial spondyloarthritis
BASDAI	Bath Ankylosing Spondylitis Disease Activity Index
BMI	body mass index
CI	confidence interval
CRP	C-reactive protein
CVD	cardiovascular disease
DAPSA	Disease Activity Index for Psoriatic Arthritis
DAS28	Disease Activity Score 28
DMARDs	disease-modifying antirheumatic drugs
EULAR	European Alliance of Associations for Rheumatology
HDL-C	high-density lipoprotein cholesterol
HR	hazard ratio
IGF-I	insulin-like growth factor-I
IL	interleukin
IL-17is	IL-17 inhibitors
ILC3	type 3 innate lymphoid cells
iNKT	invariant natural killer T cells
JAK/STATis	Janus kinase/signal transducer and activator of transcription inhibitors
LDL-C	low-density lipoprotein cholesterol
MACE	major adverse cardiovascular events
MAIT	mucosal-associated invariant T cells
MDA	minimal disease activity
MetS	metabolic syndrome
NSAIDs	non-steroidal anti-inflammatory drugs
OR	odds ratio
PASI	Psoriasis Area and Severity Index
PDE4i	phosphodiesterase-4 inhibitor
PsA	psoriatic arthritis
PsAID-12	Psoriatic Arthritis Impact of Disease-12
QoL	quality of life
RA	rheumatoid arthritis
RR	relative risk
SD	standard deviation
SDAI	Simple Disease Activity Index
TC	total cholesterol
TG	triglycerides
Th17	T helper 17
TNF-α	tumor necrosis factor alpha
TNFis	TNF-α inhibitors
T_RM_	tissue-resident memory T cells
